# Comparing the efficacy of first-line immunotherapies in autoimmune encephalitis: a scoping review

**DOI:** 10.3389/fneur.2026.1896760

**Published:** 2026-09-04

**Authors:** Rushil Pithia, John Thomas, Thomas C. Varkey

**Affiliations:** 1 College of Medicine Phoenix, University of Arizona, Phoenix, AZ, United States; 2 Banner - University Medical Center Phoenix, Phoenix, AZ, United States

**Keywords:** autoimmune encephalitis, immunotherapy, intravenous immunoglobulin, IVIG, plasma exchange, PLEX, steroids

## Abstract

**Objective:**

This scoping review aims to explore research comparing the efficacy of steroids, IVIG, and PLEX immunotherapies for autoimmune encephalitis (AE) to determine which treatment(s) lead to better clinical outcomes.

**Background:**

Autoimmune encephalitis is a neurological disease treated with several different immunotherapies, including steroids, intravenous immunoglobulin (IVIG), and plasma exchange (PLEX). Steroids are often first-line, but it is unclear whether certain immunotherapies are more clinically efficacious.

**Methods:**

A comprehensive search of the PubMed, Cochrane, and Google Scholar databases from inception to July 1, 2025, was used to identify papers using the following search terms: “autoimmune encephalitis,” “encephalitis,” “IVIG or intravenous immunoglobulin,” “PLEX or plasma exchange,” “corticosteroids,” and “monotherapy.”

**Results:**

We identified 14 publications that fell within our search criteria. 10 of the 14 papers were retrospective studies. The remaining papers included 3 prospective studies and 1 single-arm open label clinical trial. Clinical outcomes were assessed using the modified Rankin scale (mRS). At least 5 papers examined clinical outcomes in children, and all 14 papers assessed clinical outcomes in the adult population. 1 paper did not provide information on participant age ranges. 10 papers included data assessing the efficacy of steroids in AE, with 9 papers providing data that steroids improved clinical outcomes. 7 papers assessed the clinical efficacy of IVIG where 6 papers provided data on mRS improvement in AE. Finally, 3 papers assessed mRS improvement with PLEX, where all 3 papers showed PLEX led to clinical improvement. However, it should be noted that several studies had serious to critical risk of bias and moderate to high methodological concern, impeding the reliability of each study’s findings.

**Conclusion:**

There is limited data separately evaluating first-line immunotherapies to assess whether steroids, PLEX, or IVIG are clinically superior. Thus, exploring which immunotherapy correlates most strongly with improved clinical outcomes through additional prospective studies and randomized clinical trials would be beneficial. Additionally, further data on which combinations or cycling of therapies (i.e., the “zipper method”) are most clinically efficacious, depending on patient presentation, demographics, and pathogenic mechanism would be crucial in developing more comprehensive treatment protocols.

## Introduction

Autoimmune encephalitis (AE) is a neurological condition with a complex etiology that has yet to be fully understood. While there are several mechanisms that cause AE, it is generally understood that this disease results from autoantibodies attacking CNS structures, leading to associated neuroinflammation (1, 2). AE is stratified by the type of autoantibody driving the disease, as well as its classification being paraneoplastic or nonparaneoplastic, given the disease can be associated with an underlying malignancy (2). Current literature indicates that autoantibodies target either intracellular or cell surface antigens, where N-Methyl-D-Aspartate Receptor (NMDAr), a cell surface antigen, is the most commonly targeted antigen and the most well-known AE subtype (2). Other common antigens include LG1, AMPAR, GABA-A, and CASPR2, all cell-surface antigens, which cause distinct clinical presentations comprised of various neurological and motor symptoms (3).

AE symptoms typically manifest subacutely, over the course of days to weeks, and can present in a number of ways (3). Often, patients will present with fluctuations in consciousness and display a combination of neurological, psychiatric, and/or motor symptoms (3). While particular symptoms are more strongly suggestive of specific AE subtypes, their variable presentation underscores the importance of understanding AE’s diagnostic criteria (3). To diagnose AE, the patient must meet three of the following: (1) Subacute onset of memory impairment, altered mental status, or psychiatric symptoms, (2) At least one of the following: new focal CNS findings, seizures that cannot be explained by another disorder, CSF pleocytosis, or MRI features suggestive of encephalitis, and (3) Reasonable exclusion of alternative causes (more specific diagnostic criteria exists for anti-NMDAr AE) (4).

Autoantibody testing is regularly performed to categorize a patient’s AE and better guide treatment; however, treatment is typically given empirically, as prompt immunotherapy initiation is linked to better outcomes (3, 5). First-line treatment consists of immunotherapies including steroids, intravenous immunoglobulin (IVIG), and plasma exchange (PLEX) (3, 6). Depending on treatment response, patients may also require second or third-line immunotherapies or maintenance immunotherapy to improve a patient’s condition (6). Because of a lack of randomized controlled trials, current treatment strategies heavily rely on observational studies, expert opinion, and patient presentation. As a result, a vast knowledge gap exists regarding the efficacy of first-line therapies when used individually (5–7). Thus, this scoping review aims to assess the current literature on the clinical efficacy of first-line treatments in the setting of AE to determine if certain immunotherapies are clinically superior.

## Methods

To better understand the current state of scientific literature comparing the efficacy of first-line immunotherapies in AE and identify associated knowledge gaps, a scoping review was deemed most appropriate. This scoping review followed Preferred Reporting Items for Systematic Review and Meta-analyses extension for Scoping Reviews (PRISMA-ScR) guidelines (8). The review followed the framework of Arksey and O’Malley in identifying a research question, identifying relevant studies, selecting studies, recording data, and synthesizing included data (9).

Articles were assessed using the following inclusion criteria: (i) articles must be written in English; (ii) full-text copies of the studies were available via open-access or through library access via an affiliated organization; (iii) studies were written and published before July 1, 2025; (iv) studies involving patients of any age with a suspected or confirmed diagnosis of AE; (v) studies discussing or comparing the use and efficacy of first-line immunotherapies for AE; (vi) studies that used a single immunotherapy in treatment arms or treatment arms only differed by one immunotherapy; and (vii) studies used the modified Rankin Scale (mRS) as a measurement of functional outcome before and after treatment. Exclusion criteria included: (i) articles not written in English; (ii) studies not discussing AE or first-line immunotherapies; (iii) articles that were not in peer-reviewed journals; (iv) studies where full-text copies were not available via open-access or through library access via an affiliated organization, (v) studies primarily focused on second-line or long-term maintenance therapies; and (vi) studies not using mRS as a measurement of functional outcome before and after treatment.

To ensure a viable and appropriate research process, a search strategy was developed consisting of the search terms “autoimmune encephalitis,” “encephalitis,” “IVIG,” “intravenous immunoglobulin,” “PLEX,” “plasma exchange,” “corticosteroids,” and “monotherapy.” Searches were confined to articles (which included case reports, case series, clinical studies, clinical trials, observational studies, randomized controlled trials, scoping reviews, and systematic reviews) published from the inception of each database to July 1, 2025, on Google Scholar, PubMed, and Cochrane. The search was independently carried out by authors R.P. and J.T. and any disagreements in screened papers were adjudicated by author T.V. All final articles were then reviewed and the following information was extracted from each paper into Microsoft Excel including: paper title, year of publication, study design, sample size, study population, patient sex, age range, AE subtype, immunotherapy used, initial mRS, post-treatment mRS, and risk of bias.

In using Google Scholar, three unique search phrases were used: (1) “autoimmune encephalitis” AND “IVIG” AND “monotherapy,” (2) “autoimmune encephalitis” AND “PLEX” AND “monotherapy,” and (3) “autoimmune encephalitis” AND “corticosteroids” AND “monotherapy.” On PubMed, three unique search phrases were used: “encephalitis” AND “IVIG OR intravenous immunoglobulin,” (2) “encephalitis” AND “PLEX OR plasma exchange,” and (3) “encephalitis” AND “corticosteroids.” In reviewing eligible papers on PubMed, the following filters were applied: only inclusion of free full text papers which included case reports, clinical studies, clinical trials, randomized controlled trials, scoping reviews, and systematic reviews. Lastly a comprehensive search of Cochrane required the use of five unique search phrases: (1) “encephalitis” AND “corticosteroids,” (2) “encephalitis” AND “IVIG,” (3) “encephalitis” AND “intravenous immunoglobulin,” (4) “encephalitis” AND “PLEX,” and (5) “encephalitis” AND “plasma exchange.” The specific content reviewed in Cochrane included Cochrane reviews and trials.

The discrepancy in search terms used across databases is due to the more forgiving nature of Google Scholar of search parameters, thus the decision to use only “autoimmune encephalitis” as the searchable disease condition, use the abbreviated terms “IVIG” and “PLEX,” and include the term “monotherapy” was used to reduce the inherent redundancy of results. In contrast, PubMed and Cochrane provide specific search criteria that allow for more focused searches, which led to the use of more generalized and exhaustive search phrases that used “encephalitis” given that papers may have been titled or tagged with a specific antibody associated with AE instead of using the term “autoimmune encephalitis” specifically. This helped to reduce the likelihood of potential papers for this scoping review being accidentally missed. Additionally, mRS was used as a measure of clinical outcome due to its objective nature of assessing functional disability to provide a standardized method of assessing the efficacy of first-line immunotherapies across the included studies (10).

Risk of bias was assessed using design-specific instruments. Randomized studies were evaluated using RoB 2, nonrandomized comparative intervention studies using ROBINS-I, uncontrolled intervention studies using the JBI Critical Appraisal Checklist for Quasi-Experimental Studies, and case series using the JBI Critical Appraisal Checklist for Case Series. Assessments were performed for the treatment comparison, outcome, and time point relevant to the review rather than assigning a single universal judgment to each publication. JBI does not prescribe numerical scores or summary risk categories; therefore, item-level responses were reported, and the terms “moderate methodological concern” and “high methodological concern” were used as narrative summaries.

**Figure 1:**
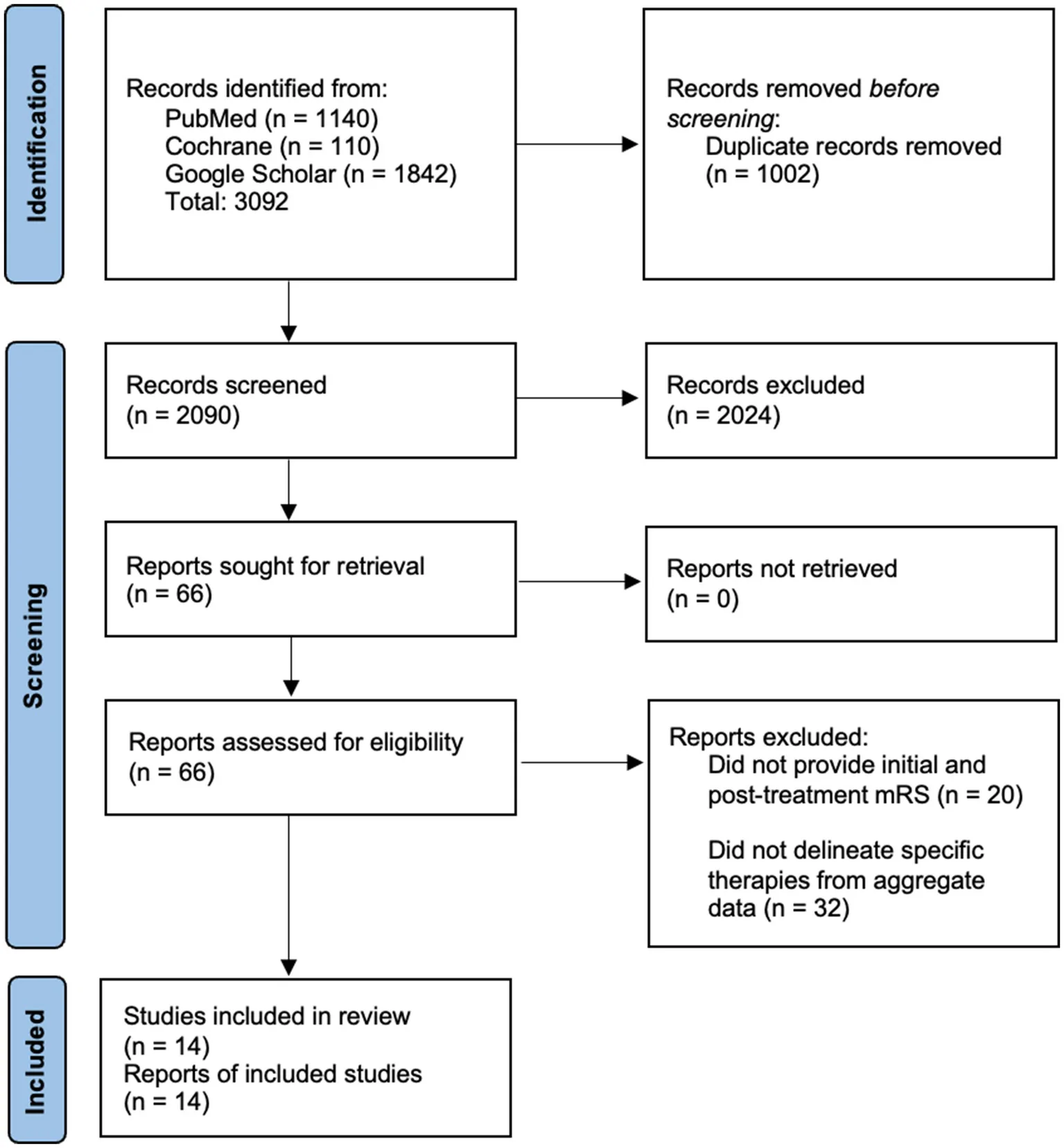
PRISMA flowchart. The PRISMA 2020 flow diagram templates are distributed in accordance with terms of the Creative Commons Attribution license: Page et al. (34). https://creativecommons.org/licenses/by/4.0/ and https://www.prisma-statement.org/prisma-2020-flow-diagram.

A PRISMA flow diagram based on the inclusion and exclusion criteria was included below (Figure 1).

## Results

A total of 3,092 articles were identified across Google Scholar, PubMed, and Cochrane. 1,002 duplicates were excluded resulting in 2090 full-text articles. These articles were reviewed based on their titles and abstracts in accordance with the exclusion criteria, resulting in 66 full-text articles. The full text of all 66 articles was reviewed and screened again alongside the exclusion criteria, excluding those which did not specify treatment groups which only received one immunotherapy and the initial and post-treatment mRS scores following immunotherapy for each patient or treatment group. The main findings of the 14 papers are summarized in Table 1. Most included studies (10/14) were retrospective studies, 3 papers were prospective studies, and 1 paper was a single-arm, open label study.

**Table 1:** Summary of characteristics of included studies.

Title	Year	Study design	Sample size	AE subtype	Population (pediatric, Adult, Both, Unspecified)	Age range	M/F counts	Immunotherapy (dosing/treatment duration, if available)	Initial treatment mRS	Post-treatment mRS	Adverse events
Rittel et al. (13)	2025	Retrospective observational study	*n* = 75	31 NMDA, 33 LG1, 11 CASPR2	Adult	NMDA (mean onset, 38), LG1/CASPR (mean onset, 63)	NMDA (9 M, 22 F); LG1/CASPR (30 M, 14 F)	ivMP (3–5 day course, 500 mg to 1,000 mg per dose)	NMDAR median mRS 3 (range 2–5), anti-LG1/CASPR2 median mRS 2.5 (range 1–5)	NMDAR median mRS 2 (range 0–5), anti-LG1/CASPR median mRS 1.75 (range 0–4)	Not specified
Shin et al. (17)	2013	Retrospective case series	*n* = 4	LG1	Adult	Mean age: 55; range: 41–66	3 M, 1 F	ivMP (500 mg or 1,000 mg for 5 days, *n* = 4)	mRS 2.25 (range 2–3)	mRS 0.5 (range 0–1)	Not specified
Yeo et al. (19)	2018	Retrospective observational study	*n* = 5	LG1, CASPR2	Adult	mean age: 65; range: 56–79	4 M, 1 F	ivMP (dosing not specified)	mRS 2.4 (range 2–4)	mrRS 1.5 (range 1–2)	Not specified
Gao et al. (12)	2016	Retrospective case series	*n* = 1	LG1	Adult	57	1 M	IV Dexamethasone (10 mg for 10 days)	3	2 (at 2 months); 1 (at 10 months)	Not specified
Reyes et al. (15)	2021	Retrospective case series	*n* = 5 (steroids), *n* = 1 (IVIG)	1 VGKC, 4 NMDA, 1 AMPAR	Both	Steroid range: 15–51; IVIG age: 7	Steroids (3 M, 3 F); IVIG (0 M, 1 F)	VGKC, NMDA, AMPAR (steroids, dosing not specified), 1 NMDA (IVIG, dosing not specified)	VGKC (mRS 5), AMPAR (mRS 1), NMDA (steroids, median mRS 2.5, range 2–3), NMDA (IVIG, mRS 5)	VGKC (mRS 5), AMPAR (mRS 5), NMDA (steroids, median mRS 1.25, range 1–2), NMDA (IVIG, mRS 0)	Not specified
Cai et al. (11)	2022	Retrospective observational study	*n* = 11 (ivMP), *n* = 11 (IVIG)	ivMP (6 NMDA, 3 LG1, 2 GABA); IVIG (6 NMDA, 2 LG1, 3 GABA)	Unspecified*	ivMP (median age: 46, IQR 26.5–51); IVIG (median age: 31, IQR 27–55.5)	ivMP (7 M, 4 F); IVIG (10 M, 1 F)	ivMP (1,000 mg or 500 mg for 3–5 days, taper with oral prednisone for 3–6 months), IVIG (2 g/kg, divided over 5 days)	ivMP (mRS 1, range 1–2); IVIG (mRS 2, range 1–3)	ivMP (4 weeks after therapy, mRS 1, range 1–2); IVIG (4 weeks after therapy, mRS 1, range 1–1.5)	Adverse drug reaction
Gong et al. (14)	2021	Prospective observational study	*n* = 37 (ivMP), *n* = 37 (IVIG)	NMDA	Both	ivMP (median age: 27, IQR: 21–36); IVIG (median age: 23, IQR: 14–35)	ivMP (16 M, 21 F); IVIG (15 M, 22 F)	ivMP (1,000 mg for 3–5 days, switch to daily prednisone 1 mg/kg first 2 weeks then taper 3–6 months); IVIG 0.4 g/kg daily for 5 days	ivMP (mRS 4.11, range 3–5); IVIG (mRS 4.2, range 3–5)	ivMP (1 month after therapy ∆mRS 2, CI 1.41–1.73); IVIG (1 month after therapy ∆mRS 1, CI 1.34–1.59)	ivMP: infusion site pain, skin rash, peripheral edema; IVIG: weight gain, infusion site pain, respiratory infection, insomnia, dyspepsia, nausea or vomiting
Melamud et al. (16)	2018	Retrospective observational study	*n* = 2 (ivMP), *n* = 2 (IVIG)	ivMP (1 NMDA, 1 LG1); IVIG (1 LG1, 1 probable AE)	Both	ivMP (mean age: 43, range 16–70); IVIG (mean age: 33.5, range 19–48)	ivMP (0 M, 2 F); IVIG (2 M, 0 F)	not specified	ivMP (mRS 4, range 3–5); IVIG (mRS 4, range 3–5)	ivMP (mRS 0.5, range 0–1); IVIG (mRS 1)	No adverse events found
Wang et al. (21)	2018	Retrospective case series	*n* = 5 (steroids), *n* = 4 (IVIG)	LG1	Adult	Steroids (mean age: 29; range: 18–44); IVIG (mean age: 48.75; range 36–66)	Steroids (2 M, 3 F); IVIG (1 M, 3 F)	Steroids, IVIG (dosing not specified)	Steroids (mRS 4, range 3–5); IVIG (mRS 3.5, range 3–4)	Steroids (2 months after therapy, mRS 2.6, range 1–4); IVIG (2 months after therapy, mRS 1.5, range 1–2)	Not specified
Rodriguez et al. (18)	2021	Retrospective case series	*n* = 49 (steroids), *n* = 21 (IVIG)	LG1	Both	Steroids (median age: 66, range 27–87); IVIG (median age: 66, range 17–79)	Steroids (37 M, 12 F); IVIG (14 M, 7 F)	ivMP (1 g 3–5 days, possible further steroid treatment); IVIG (0.4 g/kg 3–5 days, possible further infusions)	Steroids (3, range 0–5); IVIG (3, range 2–4)	Steroids (1, range 0–5); IVIG (3, range 0–3)	Steroids: insomnia, mood disturbance, weight gain; IVIG: headache, rash, fatigue
Lee et al. (20)	2022	Single-arm, open label study	*n* = 18	Not specified	Both	Unspecified**	9 M, 9 F	IVIG (0.4 g/kg/day for 5 days)	3.44 +/− 0.92	Day 8 (2.78 +/− 1.22); Day 15 (2.28 +/− 1.02); Day 29 (1.78 +/− 1.11)	Chest discomfort, diplopia, shivering
Zhang et al. (23)	2021	Prospective cohort study	*n* = 57	51 NMDA, 3 GABA, 2 LG1, 1 AMPAR	Both	median age: 26 (IQR: 21, range: 40)	30 M, 27 F	ivMP (1,000 mg or 500 mg for 3–5 days), IVIG (0.4 g/kg for 5 days), TPE (3–5 procedures per course)	Non-TPE (median mRS 4.9, range 3–5), TPE (median mRS 4.8, range 3–5)	Non-TPE at 1 month (median mRS 4.75, range 2–5), TPE at 1 month (median mRS 4.45, range 2–5); Non-TPE at 2 months (median mRS 4.46, range 2–5), TPE at 2 months (median mRS 3.88, range 1–5)	Fever, hypotension, involuntary movements, anaphylactic reaction, clot in tube
Heine et al. (24)	2016	Prospective observational case control study	*n* = 3	2 NMDA, 1 GAD	Adult	NMDA (mean age: 20, range 18–22), GAD (76)	NMDA (0 M, 2 F), GAD (0 M, 1 F)	PLEX (median: 7 sessions, range: 5–12)	NMDA (mRS 3.5, range 3–4); GAD (4)	NMDA (mRS 1.5, range 1–2), GAD (4)	Catheter-associated infection, anemia, low fibrinogen
Nieto-Aristizábal et al. (22)	2020	Retrospective observational study	*n* = 6	Not specified	Unspecified*	Not specified	Not specified	PLEX (treatment duration not specified)	4 (range 3–5)	3 (range 2–3)	Hypotension, electrolyte abnormalities, catheter-associated bleeding, catheter-associated hypersensibility

* It should be noted that a median age and interquartile range were provided which indicate at least adult participants were included, but this does not definitively rule out the inclusion of pediatric patients as well. ** A mean age of 48.8 +/− 17.4 (representing one standard deviation) was reported indicating adult patients were included, but this does not definitively rule out the inclusion of pediatric patients. M, male; F, female; mRS, modified Rankin scale; AE, autoimmune encephalitis; ivMP, intravenous methylprednisolone; IVIG, intravenous immunoglobulin; IQR, interquartile range; TPE, therapeutic plasma exchange; PLEX, plasma exchange. mRS is reported as a mean unless otherwise specified.

### Steroids

10 papers assessed the efficacy of steroids in AE, providing a pooled total of 194 patients who received only steroids in the treatment of AE. Across the 10 papers, both adult and pediatric populations were included in the chosen studies [Cai et al. (11) did not specify the age range of the study population]. Patients had a range of AE subtypes, including NMDA, LG1, CASPR2, VGKC, AMPAR, and GABA. Studies generally detailed a corticosteroid regimen of intravenous methylprednisolone at a dose of 500 mg to 1,000 mg for a typical duration of 3–5 days. Gao et al. (12) used a different steroid regimen involving a 10-day course of 10 mg of dexamethasone. 9 papers showed evidence of mRS improvement following corticosteroid treatment, however, it should be noted that studies had variability in when initial and post-treatment mRS was recorded. Rittel et al. (13) recorded initial mRS at the peak of disease in which the study does not specify if immunotherapy had already been initiated. Additionally, Gong et al. (14) recorded initial mRS at nadir of disease instead of mRS prior to immunotherapy. Regarding post-treatment mRS data, there is heterogeneity between the timeline of this endpoint and how this data was presented across studies. Certain papers such as Rittel et al. (13), Reyes et al. (15), Melamud et al. (16), and Shin et al. (17) recorded post-treatment mRS at discharge. However, Cai et al. (11) recorded post-treatment mRS at 4 weeks after immunotherapy, Gong et al. (14) recorded post-treatment mRS 1 month after immunotherapy, and Rodriguez et al. (18) recorded post-treatment mRS at first follow up which was on average 1.4 months after immunotherapy was given. Finally, Gao et al. (12) provided post-treatment mRS data at 2- and 10-months after immunotherapy while Yeo et al. (19) recorded “best” mRS after treatment without indicating a timeline of when this was recorded following immunotherapy. It should also be noted that Gong et al. (14) included ∆mRS instead of median post-treatment mRS after steroid therapy. Specific details on mRS trends are outlined in Table 1.

Adverse events from steroid treatment were recorded in 3 papers. Gong et al. (14) reported 364 events with the most common being infusion site pain, skin rash, and peripheral edema. Cai et al. (11) recorded one instance of an adverse drug reaction without specification on what the patient experienced. Finally, Rodriguez et al. (18) reported 23 adverse events which included insomnia, mood disturbance, and weight gain.

### IVIG

A total of 7 studies assessed the efficacy of IVIG in AE, with a total of 94 patients across the included studies only receiving IVIG. Both adult and pediatric populations were studied across all papers [Cai et al. (11) did not specify age range of study population]. AE subtypes included: AMPAR, NMDA, LG1, and GABA. When specified, IVIG dosing generally followed a schedule of 0.4 g/kg for 5 days, with Cai et al. (11) utilizing a regimen of 2 g/kg divided over the course of 5 days. 6 papers provided data that mRS improved with IVIG. However, as previously indicated, there were some discrepancies in when post-immunotherapy mRS was recorded across studies. Lee et al. (20) recorded post-treatment mRS at days 8, 15, and 29. Meanwhile, Cai et al. (11) recorded post-treatment mRS 4 weeks after therapy, Gong et al. (14) recorded post-treatment mRS 1 month after therapy, and Wang et al. (21) recorded post-treatment mRS 2 months after therapy. Additionally, Gong et al. (14) did not include median post-treatment mRS and instead included ∆mRS after therapy. Specific details on mRS trends are outlined in Table 1.

Certain papers compared the efficacy of steroids and IVIG which led to a heterogeneity of findings. Cai et al. (11) provided statistical data comparing the distribution of clinical improvement in NMDA AE patients by assessing post-treatment mRS and found that there was no significant difference in clinical outcomes between treatment groups (*p* = 0.587). Gong et al. (14) compared the time to reduce mRS by at least 1 point between steroids and IVIG in patients with NMDA AE, indicating that the time to reduce mRS was slightly shorter in the IVIG group although this was not statistically significant. Rodriguez et al. (18) assessed mRS improvement in patients with LG1 AE who received either steroids or IVIG and found significant improvement in the steroid group at first follow-up which was on average 1.4 months (*p* = 0.004). It should be noted that long-term outcomes (appraised via mRS scores) which was specified as > 24 months after initial presentation were not statistically significant between treatment arms (*p* = 0.45) (18).

Adverse events were recorded in 3 papers. Lee et al. (20) included a total of 36 adverse events, with 5 recorded events being possibly or likely related to infusion. This included chest discomfort, diplopia, and shivering. Gong et al. reported 215 adverse events related to IVIG treatment, with the most common including weight gain, infusion site pain, respiratory infection, insomnia, dyspepsia, and nausea or vomiting. Lastly, Rodriguez et al. (18) recorded 5 adverse events in their IVIG treatment group which included headache, rash, and fatigue.

### PLEX

3 studies provided data on the efficacy of PLEX in AE, with an aggregate of 66 patients being included across all papers. Across two studies, both adult and pediatric populations were assessed while Nieto-Aristizábal et al. (22) did not specify the age range of their study population. AE subtypes across all papers included: NMDA, GABA, LG1, AMPAR, and GAD. PLEX regiments differed across each paper where Zhang et al. (23) provided 3–5 procedures per course, Heine et al. (24) had participants complete a range of 5–12 sessions, and the last study did not include specific details on the number of PLEX sessions provided. Each paper provided data that mRS improved with PLEX, however the time at which post-treatment mRS was recorded differed per paper. Specifically, Zhang et al. (23) recorded post-treatment mRS at the 1- and 2-month mark while other studies provided post-treatment mRS shortly after the conclusion of PLEX treatment. Specific details on mRS trends are outlined in Table 1.

Zhang et al. (23) indicated that the PLEX group exhibited greater mRS improvement at the 1- and 2-month mark (*p* = 0.03 and *p* = 0.01 respectively). However, in comparing these outcomes between treatment arms, mRS improvement at the 2-month mark appears more clinically significant compared to the 1-month mark. It should be noted that in study by Zhang et al. (23), both treatment arms also received steroids and IVIG, which may confound the conclusions of this paper. The study was ultimately included given the clear study design where PLEX was the only difference in treatment arms which allowed the treatment outcomes to largely be attributed to PLEX treatment.

Nieto-Aristizábal et al. (22) concluded that the change in mRS in the PLEX group was statistically significant, however the reported *p*-value was 0.0516, which is above the generally used cutoff for statistical significance (*p* = 0.05).

Regarding adverse events, Zhang et al. (23) reported 91 events including fever, hypotension, involuntary movement, anaphylactic reaction, and a clot in the catheter. Heine et al. (24) noted 3 adverse events: catheter-associated infection, anemia, and low fibrinogen. Lastly, Nieto-Aristizábal et al. (22) noted 10 adverse events including hypotension, electrolyte abnormalities, catheter-associated bleeding, and catheter associated hypersensibility.

In assessing the risk of bias, five nonrandomized comparative studies were analyzed using ROBINS-I and were judged to be at critical risk of bias at the longest follow-up, primarily because of confounding by indication, post-treatment exclusions, treatment switching or escalation, baseline severity differences, and incomplete follow-up. Rodriguez et al. (18) provided the most informative acute comparison of corticosteroids and IVIG and was rated serious risk initially, but critical risk at long-term follow-up because fewer than half of eligible patients remained and subsequent immunotherapies were common. Heine et al. (24) was rated high risk using RoB 2 because randomization and allocation concealment were inadequately described, treatment groups were clinically imbalanced, and mRS assessment was unblinded. Two uncontrolled intervention studies assessed with the JBI quasi-experimental checklist lacked concurrent controls and therefore could not isolate the effects of IVIG or plasma exchange, while four case series provided primarily descriptive evidence because of small samples, heterogeneous treatment pathways, and the absence of appropriate comparison groups. An aggregate risk of bias summary is included in Table 2.

**Table 2:** Risk of bias summary for included studies.

Study	Design and comparison	Tool	Outcome/time point	Overall appraisal	Primary concerns	Contribution to the review
Rittel et al., 2025 (13)	Multicenter nonrandomized comparison of IVIG- and PE-containing first-line strategies	ROBINS-I	mRS at 12 months	Critical risk	Serious confounding by indication and highly incomplete, selected 12-month follow-up	Comparative observational evidence; interpreted cautiously
Shin et al., 2013 (17)	Retrospective nonrandomized comparison of initial corticosteroid monotherapy versus corticosteroids plus IVIG in 12 evaluable patients	ROBINS-I	Final mRS and relapse over 1–24 months	Critical risk of bias	Uncontrolled confounding, highly variable treatment timing and follow-up, response-driven addition of IVIG, PLEX and second-line therapies, retrospective unblinded outcomes, and *post hoc* exclusion of a poor-outcome combination-therapy patient that changed the treatment association from nonsignificant to significant	Direct but critically biased comparative evidence; hypothesis-generating only
Yeo et al., 2018 (19)	Diagnostic cohort with a small LGI1/CASPR2 treatment subgroup	JBI case series	Best mRS and relapse	Moderate methodological concern*	Mixed neurological syndromes and no clinically appropriate treatment comparator	Contextual/descriptive evidence
Gao et al., 2016 (12)	Retrospective LGI1 case series	JBI case series	mRS at 10 months	Moderate methodological concern*	Ten patients, treatment switching, unadjusted analyses, and unclear complete inclusion	Descriptive evidence
Reyes et al., 2021 (15)	Retrospective case series	JBI case series	Discharge mRS and mortality	Moderate methodological concern*	Uncertain consecutive inclusion, heterogeneous treatments, and no comparative analysis	Descriptive evidence
Cai et al., 2022 (11)	Retrospective IVMP versus IVIG versus IVMP plus IVIG comparison	ROBINS-I	Favorable mRS at 1 year	Critical risk	Patients requiring additional IVIG, second-line therapy, or maintenance treatment were excluded after initial treatment	Comparative observational evidence; likely enriched for treatment responders
Gong et al., 2022 (14)	Prospective registry comparison of IVIG, IVMP, and combination therapy	ROBINS-I	mRS at 12 months and relapse	Critical risk	Post-treatment exclusions, switching between therapies, duplicate treatment observations, and incomplete handling of deaths	Comparative observational evidence; association only
Melamud et al., 2020 (16)	Retrospective case series	JBI case series	Final mRS and relapse	Moderate methodological concern*	Ten patients, heterogeneous treatment pathways, and follow-up ranging from 5 to 116 months	Descriptive evidence
Wang et al., 2018 (21)	Retrospective nonrandomized comparison of corticosteroids, IVIG and corticosteroids plus IVIG	ROBINS-I	mRS and BPRS at 2 months	Serious risk of bias	Unexplained treatment allocation, no adjustment for baseline severity or treatment delay, poorly described treatment protocols, retrospective unblinded outcome assessment and extremely small treatment groups	Small direct comparative study; observed IVIG-associated outcomes are hypothesis-generating
Rodriguez et al., 2022 (18)	Retrospective corticosteroid versus IVIG monotherapy comparison	ROBINS-I	Acute first follow-up	Serious risk	Nonrandom treatment selection, heterogeneous regimens, variable follow-up timing, and unblinded assessment	Most informative acute observational comparison
Rodriguez et al., 2022 (18)*	Same cohort	ROBINS-I	≥24-month outcomes	Critical risk	Fewer than half of eligible monotherapy patients contributed and subsequent immunotherapies were common	Long-term effect of initial therapy not reliably estimable
Lee et al., 2022 (20)	Prospective single-arm IVIG study	JBI quasi-experimental	mRS at day 29	High methodological concern*	No comparator, recent corticosteroid exposure, unblinded assessment, and frequent rescue immunotherapy	Uncontrolled treatment-trajectory evidence
Zhang et al., 2021 (23)	Prospective nonrandomized TPE-containing versus non-TPE rescue strategies	ROBINS-I	mRS at 12 months	Critical risk	Non-TPE allocation partly determined by hypotension, infection, behavioral disturbance, or treatment refusal	Comparative observational evidence; not considered a reliable causal estimate
Heine et al., 2016 (24)	Prospective pilot comparison of immunoadsorption and PE with stated random allocation	RoB 2	Immediate post-pheresis mRS	High risk	Randomization and concealment were inadequately described, groups were imbalanced, and outcome assessment was unblinded	Comparative pilot evidence; equivalence not established
Nieto-Aristizábal et al., 2020 (22)	Retrospective uncontrolled TPE study; six AE patients	JBI quasi-experimental	Post-TPE mRS	High methodological concern*	No control group, retrospective mRS reconstruction, uncertain co-interventions, and incomplete follow-up	Uncontrolled descriptive evidence

JBI does not prescribe numerical scores or official low/moderate/high categories. *Rodriguez et al. (18) was included twice to assess risk of bias associated with acute and long-term outcomes. “Moderate methodological concern” and “high methodological concern” are review-defined summary descriptions.

## Discussion

This scoping review reveals a lack of data comparing the independent clinical efficacy of individual first-line immunotherapies in autoimmune encephalitis. This limitation reflects both weaknesses in study design and how the clinical landscape commonly incorporates early multimodal treatment adjusted to disease severity, particularly in patients presenting with anti-NMDA encephalitis (24).

Across the included studies, corticosteroids had the largest and most consistent evidence base, whereas evidence for IVIG and especially PLEX was smaller and less direct. Nine of the ten studies evaluating corticosteroid monotherapy reported improvement in mRS with favorable outcomes observed across anti-NMDA, anti-LGI1, and anti-CASPR2 encephalitis. IVIG was also associated with improvement in six of seven of the evaluating studies, but direct comparisons with corticosteroids produced heterogeneous results. In predominantly anti-NMDA populations, Cai et al. (11) found no significant difference in functional outcomes between IVIG and intravenous methylprednisolone. Similarly, Gong et al. (14) found no statistically significant difference in the time required to achieve mRS improvement. In contrast, Rodriguez et al. (18), who conducted the largest acute comparison in anti-LGI1 encephalitis, demonstrated significantly greater early improvement with corticosteroids than with IVIG. However, a smaller study by Wang et al. (21) showed numerically better two-month functional outcomes among anti-LG1 AE patients receiving IVIG. Although corticosteroids demonstrated the clearest observed advantage in anti-LGI1 encephalitis, these findings suggest that neither corticosteroids nor IVIG can be considered universally superior across all forms of AE. The evidence for PLEX was more limited because only three studies met the inclusion criteria, treatment protocols varied substantially, and PLEX was generally used after other immunotherapies or as part of multimodal regimens. Zhang et al. (23) observed that the addition of PLEX to corticosteroids and IVIG was associated with greater functional improvements at 1 and 2 months, suggesting that PLEX may accelerate earlier recovery in patients with severe disease. In contrast, Nieto-Aristizábal et al. (22) reported only a modest mRS reduction that failed to meet statistical significance. The available evidence therefore supports PLEX as a potentially valuable adjunct in particularly severe or incompletely responsive AE but does not establish its superiority as an isolated first-line treatment.

Differences among AE subtypes were also apparent, though the strength of the evidence varied greatly. In anti-NMDAR encephalitis, corticosteroids and IVIG were both associated with functional improvement, but the direct comparisons that were available did not consistently favor either treatment. PLEX appeared most promising as an adjuvant therapy in this subtype, particularly in the severe AE population studied by Zhang et al. (23), where anti-NMDAR encephalitis accounted for most of their participants. This observation aligns with current practice favoring combined first-line treatment in severe anti-NMDA encephalitis rather than reliance on a single agent. Anti-LGI1 encephalitis demonstrated the strongest subtype-specific treatment response. Several studies showed substantial improvement after corticosteroids, but Rodriguez et al. (18) found significantly greater early functional and seizure-related improvement with corticosteroids than with IVIG. Although Wang et al. (21) reported numerically better outcomes with IVIG, its treatment groups contained a much smaller number of patients and allocation was not adjusted for baseline differences. Therefore, the greater weight of evidence in this review favors corticosteroids as a particularly effective acute treatment for anti-LGI1 encephalitis. This conclusion is also consistent with more recent observational evidence associating acute methylprednisolone treatment with more favorable long-term outcomes in this subtype (25). In this review, evidence for anti-CASPR2, AMPAR, and GABA receptor encephalitis was too sparse and often embedded within mixed cohorts to support any treatment-specific conclusions. Similarly, the absence of improvement in the single PLEX-treated patient with anti-GAD antibodies in this study specifically is insufficient to establish reduced PLEX responsiveness for this AE subtype. The current literature evaluating the efficacy of PLEX between different antibody AE subtypes remains limited, but generally suggests greater responsiveness from subtypes associated with neuronal cell surface antibodies including NMDA, LG1, and CASPR2, compared to intracellular or intracellular-synaptic antigens, such as anti-Hu and GAD65 (26). Overall, the findings suggest that immunotherapy response may differ according to antibody subtype, making it difficult to allow for cross-study comparisons. Future studies with adequate power and subtype isolation are needed before the superiority of a single treatment can be established.

As previously stated, the overall pattern observed in this review is consistent with current clinical practice, in which corticosteroids, IVIG, and PLEX are all considered first-line immunotherapies that are frequently administered in sequence or in combination rather than as independent monotherapies. Broader expert recommendations similarly support the addition of IVIG or PLEX when symptoms are severe, corticosteroids are contraindicated, or there is a poor initial response. This practice pattern helps explain why relatively few studies in the present review were able to isolate the effect of a single therapy. However, the absence of supporting evidence for a superior treatment across the included studies should not be interpreted as evidence that the three therapies are interchangeable. Rather, these findings support that treatment selection is influenced by disease severity, antibody subtype, comorbidities, contraindications, treatment availability, and need for escalation.

When comparing treatment response across population types from the included studies, no clear trends can be observed in terms of treatment responsiveness across adult or pediatric populations. Analyzing overall trends between population types is largely limited by the small pooled sample size across these studies where only 5 studies definitively included data on pediatric patients. Amongst these 5 studies, only ivMP and IVIG monotherapies were used, thus comparing treatment response to PLEX in the adult versus pediatric population cannot be assessed. Explicit evidence on differences in treatment response between adult and pediatric populations remain sparse, but studies do indicate that older populations (>65) are more likely to use second line immunotherapies, in addition to requiring ICU admission and mechanical ventilation (27). Ultimately, generating more focused studies that stratify treatment response based on age may be a future area of study considering the incidence of autoimmune encephalitis appears comparable in both the pediatric and adult populations (28, 29).

While this scoping review followed a structured methodology, several limitations should be considered. Study selection and data abstraction may have been susceptible to reviewer bias. The available evidence was also limited by the small number of comparative studies, heterogeneity in follow-up intervals, and several methodological concerns across the literature, including confounding by indication, incomplete follow-up, and absence of appropriate controls. These limitations prevent the conclusion of causal evidence and the reliable comparison of the independent effects of individual first-line immunotherapies. Additionally, this study only assessed three databases, where other databases such as Embase (which could have included eligible papers not found elsewhere) were not utilized due to a lack of institutional access. One of these databases, Google Scholar, while useful in assessing grey literature, has inherent weaknesses in building comprehensive search sets and has unknown updating practices which affect the database’s reliability and has inherent impacts on the reproducibility of this study (30, 31). Also, using only papers written in English that were free full-articles available through open or institutional access introduced availability bias, leading to yet another cause of eligible studies being potentially omitted from this paper. Again, it should also be noted that the serious to critical risk of bias and moderate to high methodological concern across several of the included studies limits the generalizability of this study and thus observed outcomes should be analyzed with caution.

Overall, these studies discuss a heterogeneity of findings which make it difficult to determine which immunotherapies are more efficacious for AE, which is largely due to how relatively rare of a disease it is, with an incidence of 13.7/100,000, which limits sample size (29). Additionally, each study varies in study design and primary outcomes which limits head-to-head comparison and identifying overall trends of immunotherapy efficacy based on aggregate data. Current treatment guidelines for AE are based largely on retrospective studies, thus the development of clinical trials to effectively test the clinical efficacy of immunotherapies is warranted to develop a better framework for how AE treatment should be approached (13, 32). Newly emerging treatment regimens for autoimmune diseases include the “zipper method” which involves alternating between cycles of PLEX and IVIG, typically over the course of several days (33). Although much of the current literature focuses on treating other diseases such as Guillain-Barré syndrome and acute disseminated encephalomyelitis, the zipper method can likely be extended to AE management and serves as a potential avenue for further research to evaluate for improved clinical outcomes (33). A potential randomized controlled trial in autoimmune encephalitis patients to evaluate the zipper method could be run with patients being randomized based off the current recommendations for treatment. In those patients who meet criteria for treatment based on the APE2 score and RITE2 scores these patients are empirically treated with immunotherapies at the time of likely diagnosis. In this trial, patients would be randomized to either PLEX, IVIG, or the Zipper Method in a 1 to 1 to 1 ratio with each of the patients being treated with steroids as is standard in the treatment of this entity. The zipper method consists of a round of treatment with PLEX followed by a standard dose of IVIG immediately following the PLEX session. The PLEX sessions are done with 48 h between each session to minimize the risk of significant bleeding, with a secondary benefit in the zipper method allowing 48 h for the IVIG to have an immunomodulatory effect. Such a trial would allow for IVIG and PLEX to be evaluated against each other and allow for the zipper method to be evaluated against both individual treatment modalities. Subgroup analysis could be run depending on the number of enrolled patients as the antibodies become available, however, randomization via the causal antibody would not be possible as current recommendations do not recommend waiting for confirmatory testing before starting treatment due to the concern for potential permanent brain injury and death. Until such a trial can be run to definitively answer which of the major immunotherapies provides the best treatment option, the treatment for AE remains fraught for optimization as differing AE pathophysiology and patient presentation create unique challenges requiring therapeutic customization to ensure the best possible patient outcomes.

## Data Availability

The original contributions presented in the study are included in the article/supplementary material, further inquiries can be directed to the corresponding author.
